# NF-kB2 Genetic Variations are Significantly Associated with Non-Small Cell Lung Cancer Risk and Overall Survival

**DOI:** 10.1038/s41598-018-23324-3

**Published:** 2018-03-27

**Authors:** Foteinos-Ioannis D. Dimitrakopoulos, Anna G. Antonacopoulou, Anastasia E. Kottorou, Stella Maroussi, Nikolaos Panagopoulos, Ioulia Koukourikou, Chrisoula Scopa, Melpomeni Kalofonou, Angelos Koutras, Thomas Makatsoris, Helen Papadaki, Dimitrios Dougenis, Malcolm Brock, Haralabos P. Kalofonos

**Affiliations:** 1 Molecular Oncology Laboratory, Division of Oncology, Department of Internal Medicine, Medical School, University of Patras, Patras, Greece; 2 “G. Gennimatas” General Hospital of Athens, Neurology Department, Athens, Greece; 3 Department of Cardiothoracic Surgery, Medical School, University of Patras, Patras, Greece; 4 Department of Pathology, Medical School, University of Patras, Patras, Greece; 5 Institute of Biomedical Engineering, Imperial College London, London, United Kingdom; 6 Department of Anatomy, Medical School, University of Patras, Patras, Greece; 7 Division of Thoracic Surgery, Department of Surgery, Johns Hopkins University School of Medicine, Baltimore, MD USA

## Abstract

During the last decade, a growing number of publications implicate NF-kB2 in NSCLC pathogenesis. Here, we investigated the clinical relevance of *NF-kB2* single nucleotide polymorphisms (SNPs) rs7897947, rs11574852 and rs12769316 in NSCLC and their association with NF-kB2 protein and mRNA levels. Our data show that TT (rs7897947T >G**)** and AA (rs12769316G >A) genotypes were strongly associated with an increased risk for NSCLC (P = 0.019 and P = 0.003, respectively). Additionally, in multivariate analysis, TT (rs7897947T >G**)** homozygosity was associated with worse 2- and 3-year survival rates (P = 0.030 and P = 0.028, respectively), especially among patients with stages III/IV, who had worse 2, 3 and 5-year survival (P = 0.001, P = 0.022 and P = 0.035, respectively). In chemotherapy-treated patients, TT (rs12769316G >A) homozygosity was also associated with worse 2- and 3-year survival compared to G allele carriers (P = 0.006 and P = 0.014, respectively). Furthermore, rs12769316 was correlated with survival outcome of stage I and II patients (P = 0.031 and P = 0.006, respectively). Interestingly, amongst the patients who developed metastases, A allele carriers had better 5-year survival (P = 0.020). In addition, rs12769316 was associated with NF-kB2 protein (P = 0.001) and mRNA expression (P = 0.017) as well as with tumor maximum diameter (P = 0.025). Overall, this study suggests that rs7897947 and rs12769316 are involved in NSCLC susceptibility, in treatment response and in clinical outcome.

## Introduction

More than one million deaths per year are caused by lung cancer, making it the most frequently fatal cancer type in the western world^1^. Although current knowledge on the pathobiology of the disease has impressively expanded in the last decades, the survival rates of lung cancer patients have seen little improvement^2,3^. In the foreseeable future, the problem is expected to augment at a global level due to the increase in cigarette smoking, air pollution (indoor or outdoor) and occupational exposure to hazardous factors especially in less developed countries^1,4^. Non-small cell lung cancer (NSCLC) is the major histological subtype (adenocarcinomas, squamous cell carcinomas, large cell carcinomas) of epithelial lung malignancies, linked to about 80–85% of all lung cancer cases^5^.

Although a great variety of genes and signalling pathways have been implicated in the development and progression of human lung cancer, the role of nuclear factor kappa-light-chain-enhancer of activated B cells (NF-kB) has become an object of intense study in epithelial lung malignancies^6–8^ only in the last decade^6–8^. This transcription factor has been characterized as a “double-edged sword” since, on the one hand, its role is important for the immune response against cancer and on the other hand, under specific conditions, its activation can promote inflammation and tumor development^9^.

**Figure 1:**
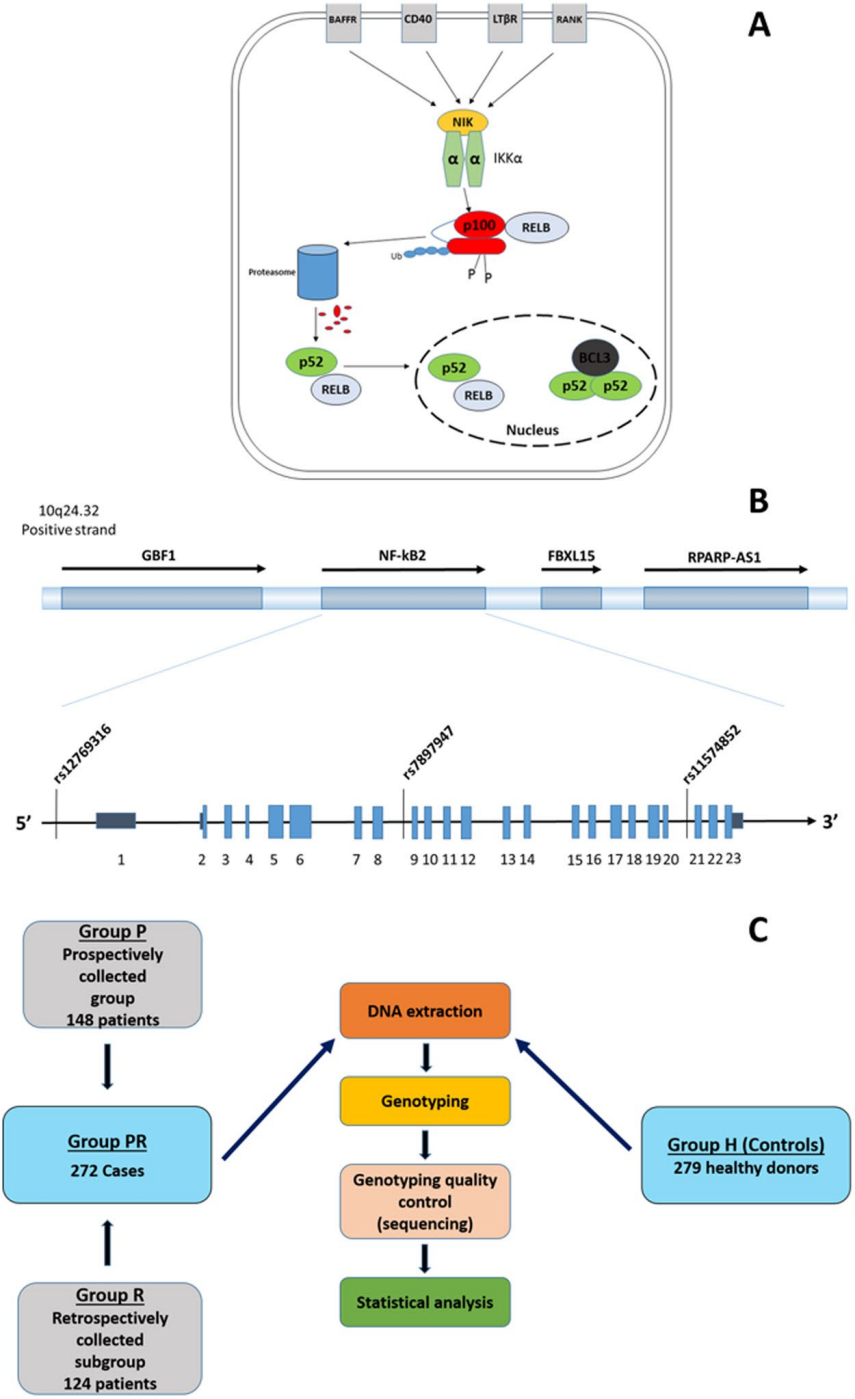
(**A**) Schematic diagram of the alternative pathway of NF-kB (Reprinted from *Lung Cancer*, 89, 3, Dimitrakopoulos *et al*., 311–319, Variant of BCL3 gene is strongly associated with five-year survival of non-small-cell lung cancer patients, 2015, with permission from Elsevier). (**B**) Genomic positions of studied SNPs in *NF-kB2* gene. (**C**) The study design outline. Abbreviations: BAFFR; tumor necrosis factor receptor superfamily member 13 C, CD40; CD40 molecule, TNF receptor superfamily member 5, LTβR; Lymphotoxin Beta Receptor (TNFR Superfamily, Member 3), RANK; Receptor Activator Of Nuclear Factor-Kappa B, NIK; NF-Kappa-Beta-Inducing Kinase, IKKα; IκB Kinase α, p100; nuclear factor NF-kappa-B p100 subunit, p52; nuclear factor NF-kappa-B p52 subunit, RELB; Transcription factor RelB, BCL3; B-Cell CLL/Lymphoma 3, FBXL15; F-Box and Leucine-Rich Repeat Protein 15, GBF1; Golgi Brefeldin A Resistant Guanine Nucleotide Exchange Factor 1, NF-kB2; nuclear factor-kappa B2, RPARP-AS1; RPARP antisense RNA 1.

The NF-kB family consists of seven members [p105/p50 (NF-kB1), p100/p52 (NF-kB2), p65 (RelA), RelB, c-Rel], which are encoded by five genes (*NF-kB1, NF-kB2, RELA, RELB, c-REL*)^10,11^. *NF-kB1* and *NF-kB2* encode the precursor proteins p105 and p100. Subsequently, these molecules are proteasomally cleaved, leading to the production of functional proteins, p50 and p52, respectively^11,12^. The NF-kB family members constitute two distinct major signaling pathways, the “classical” and the “alternative”^13^. In the alternative pathway, two of the central players are p100/p52 and RelB, which together give rise to a transcriptionally active heterodimer (Fig. 1A).

Although the role of the classical pathway in the initiation and development of NSCLC has been well documented, the involvement of the alternative pathway of NF-kB has been described relatively recently^14–17^. Our group has shown that the transcription factor NF-kB (p100/p52) is overexpressed in NSCLC, with NF-kB2 immunopositivity being associated with regional lymph node infiltration, tumor stage and tumor grade^15^.

Previous reports have documented the role of single nucleotide polymorphisms (SNPs) of NF-kB in a great variety of diseases^18,19^. In particular, genetic variations of critical components of the classical NF-kB pathway have been associated with an increased risk for cancer development, as well as with playing a significant role in the clinical outcome and patients’ response to treatment^19–28^. However, less is known about the role of *NF-kB2* SNPs in cancer in general, and in lung cancer, in particular^20,23,29^. Recently, our group demonstrated a strong association of the tagging-SNP rs8100239 of the *BCL3 (*B-Cell Leukemia/Lymphoma 3) gene, a major transcriptional regulator of the NF-kB pathway, with survival outcome of NSCLC patients^14^. This suggests an involvement of NF-KB family members’ SNPs in NSCLC pathobiology.

In this context, the aim of our study was to investigate the association of three *NF-kB2* SNPs (Fig. 1B; rs7897947, rs11574852 and rs12769316) with NF-kB2 protein and mRNA levels, NSCLC risk and clinical outcomes of NSCLC patients. The selection was based on existing literature and tag SNP data obtained with internet-based tools. Rs12769316 is located on the promoter region of the *NF-kB2* gene, while rs7897947 and rs11574852 are located on introns 8 and 20, respectively. To our knowledge, this is the first study whereby the role of genetic variations of *NF-kB2* is being assessed in lung cancer.

## Results

### *NF-kB2* SNP rs7897947 Was Associated With Increased NSCLC Risk

Genotype distributions for rs7897947 were significantly associated with the development of NSCLC. Although no difference was observed in allele specific frequencies between NSCLC patients and healthy controls, an increased risk for NSCLC was noted for the rs7897947 TT genotype compared to the G allele carriers (Table 1).

**Table 1:** ORs and 95% CIs for NSCLC in relation to genotypes of studied *NF-kB2* SNPs.

SNP	Counts		χ^2^	Univariate analysis^b^	
Genotype	Cases n (%)	Controls n (%)	*P* ^a^	*P*	OR (95% CI)
Total	268	279	—	—	—
**rs7897947**	264	279	—	—	—
ΤΤ	145 (54,9)	125 (44,8)	0.052	—	1.000
GT	89 (33,7)	120 (43,0)		**0.016**	0.639 (0.444–0.920)
GG	30 (11,4)	34 (12,2)		0.326	0.761 (0.441–1.313)
ΤΤ + GΤ vs GG	234 (88,6) 30 (11,4)	245 (87,8) 34 (12,2)		0.766	1.082 (0.642–1.825)
ΤΤ vs GT + GG	145 (54,9) 119 (45,1)	125 (44,8) 154 (55,2)		**0.019**	1.501 (1.070–2.106)
T allele	379 (71.8)	370 (66.3)	0.051	0.052	1.292 (0.998–1.673)
G allele	149 (28.2)	188 (33.7)			0.774 (0.598–1.002)
**rs12769316**	242	278	—	—	—
AA	20 (8.3)	7 (2.5)	**0.012**		1.000
AG	62 (25.6)	71 (25.5)		**0.005**	0.280 (0.116–0.679)
GG	160 (66.1)	200 (71.9)		**0.012**	0.306 (0.121–0.771)
AA + AG vs GG	82 (33.9) 160 (66.1)	78 (28.1) 200 (71.9)		0.151	1.314 (0.905–1.909)
AA vs AG + GG	20 (8.3) 222 (91.7)	7 (2.5) 271 (97.5)		**0.005**	3.488 (1.448–8.399)
A allele	102 (21.1)	85 (15.3)	**0.015**	**0.016**	1.480 (1.077–2.033)
G allele	382 (78.9)	471 (84.7)			0.676 (0.492–0.929)
**rs11574852**	261	278	—	—	—
AA	239 (91.6%)	254 (91.4%)	0.624	—	1.000
AC	22 (8.4%)	23 (8.3%)		0.999	0.984 (0.534–1.812)
CC	0 (0.0%)	1 (0.4%)		—	—
A allele	0,9579	0,9585	0.821	0.821	1.070 (0.596–1.922)
C allele	0,0421	0,0415			0.935 (0.520–1.679)

^a^*P* derives from the χ^2^ test and refers to overall association of genotypes with NSCLC risk. ^b^P, OR and 95% CI derived from logistic regression analysis. Abbreviations: CI, confidence interval; OR, odds ratios.

#### *NF-kB2* SNP rs7897947 Was Associated With Overall Survival

**Figure 2:**
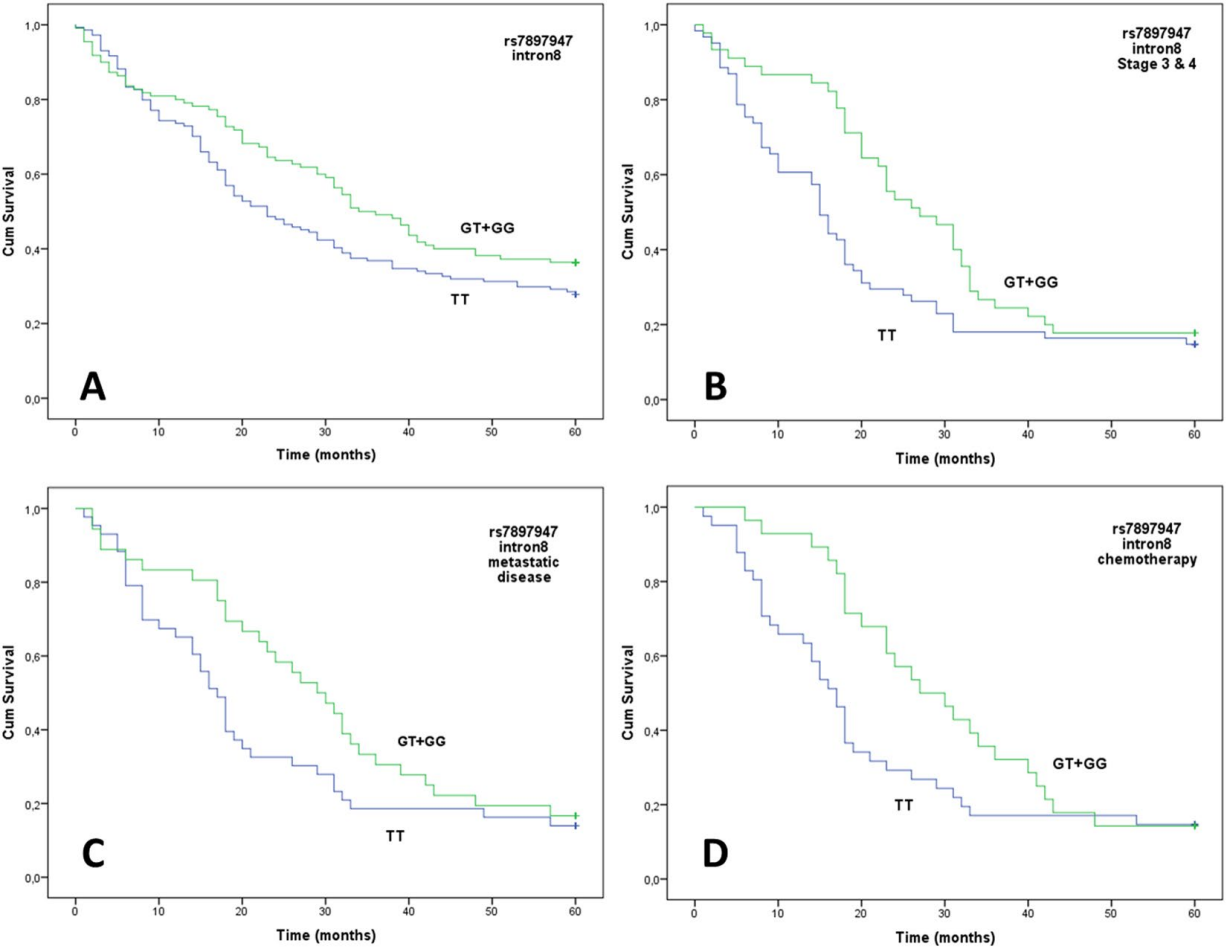
Overall survival (OS) and rs7897947 genotypes. Kaplan-Meier plot of OS according to the dominant model for the variant allele (G). (**A**) all patients, (**B**) patients of stage 3 and 4, (**C**) patients who developed metastatic disease in brain, bones, adrenals and liver, (**D**) patients who received chemotherapy.

Among all NSCLC cases, the rs7897947 TT homozygotes were associated with poorer OS compared to G allele carriers after two and three years of observation on univariate analysis with this effect being more prominent after the first 6 months of observation when the survival curves begin to separate (Table 2; Fig. 2A). Prognostic significance for 2- and 3-year OS persisted in multivariate analyses adjusted for age, sex, primary site, stage, and histological subtypes (Table 3). The same trend was also observed after 5 years of follow-up monitoring in both univariate and multivariate analyses, albeit without reaching statistical significance (Tables 2 and 3).

**Table 2:** Univariate analysis of dominant and recessive models of rs7897947, rs12769316 and rs11574852 with the OS of NSCLC patients.

SNP	Base change	Univariate analysis 2-year OS Log-rank P HR, 95% CI			Univariate analysis 3-year OS Log-rank P HR, 95% CI			Univariate analysis 5-year OS Log-rank P HR, 95% CI		
Rs number	ancestral >derived	General	Dominant	Recessive	General	Dominant	Recessive	General	Dominant	Recessive
rs7897947	T > G	**0.030** —	**0.013** 1.618 (1.102–2.377)	0.894 1.041 (0.572–1.893)	0.102 —	**0.033** 1.428 (1.023–1.994)	0.357 1.314 (0.728–2.372)	0.163 —	**0.062** 1.329 (0.981–1.801)	0.294 1.323 (0.778–2.247)
rs12769316	G > A	0.891 —	0.632 1.101 (0.738–1.644)	0.866 1.060 (0.535–2.099)	0.992 —	0.967 0.993 (0.696–1.416)	0.900 0.962 (0.519–1.781)	0.682 —	0.513 1.116 (0.800–1.557)	0.421 0.781 (0.423–1.440)
rs11574852	A > C	0.335 —	—	—	0.961 —	—	—	0.750 —	—	—

Rs7897947 dominant: TTvsGT + GG; rs7897947 recessive: TT + GTvsGG; rs12769316 dominant: AA + GAvsGG; rs12769316 recessive; AAvsGA + GG.

**Table 3:** Multivariate analysis of OS of NSCLC patients according to genotypes of polymorphisms rs7897947, rs12769316 and rs11574852.

Polymorhism	Base change	Multivariate analysis 2-year OS		Multivariate analysis 3-year OS		Multivariate analysis 5-year OS	
		HR (95% CI)	Cox proportional Hazard models *P*	HR (95% CI)	Cox proportional Hazard models *P*	HR (95% CI)	Cox proportional Hazard models *P*
rs7897947 dominant (TTvsGT + GG) recessive (TT + GTvsGG)	T > G	1.591 (1.045–2.423) 0.826 (0.419–1.630)	**0.030** 0.582	1.503 (1.044–2.165) 1.147 (0.590–2.231)	**0.028** 0.685	1.360 (0.977–1.893) 1.202 (0.669–2.161)	0.069 0.538
rs12769316 dominant (AA + GAvsGG) recessive (AAvsGA + GG)	G > A	1.195 (0.773–1.849) 1.391 (0.689–2.808)	0.423 0.358	1.103 (0.750–1.622) 1.324 (0.702–2.495)	0.618 0.386	0.974 (0.678–1.400) 1.025 (0.548–1.919)	0.887 0.937
rs11574852 (AAvsAC)	A > C	1.484 (0.645–3.411)	0.353	0.849 (0.604–2.126)	0.609	1.041 (0.558–1.942)	0.899

Covariates: Age, sex, primary site, stage, histological subtype.

Further analysis by stage stratification revealed a prognostic value for the rs7897947 for 2-, 3- and also 5-year survival for patients of stages III and IV (P = 0.001; HR, 2.271; 95% CI, 1.358–3.799, P = 0.022; HR, 1.629; 95% CI, 1.056–2.514 and P = 0.035; HR, 1.551; 95% CI, 1.019–2.361, respectively). In particular, the prognosis of the TT patients was worse compared to the G allele carriers (Fig. 2B). In contrast, survival of stage I or II patients was unaffected by rs7897947 (P = 0.624; HR, 0.851; 95% CI, 0.444–1.631 and P = 0.109; HR, 1.702; 95% CI, 0.874–3.313 for the 5-years OS).

Moreover, the TT genotype was correlated with a worse 2- and 3-year OS, compared to the presence of the G allele in 79 patients who developed metastases in liver, brain, bones and/or adrenals although significance remained for the 2-year OS after FDR analysis and was marginally lost for the 3-year survival (Fig. 2C; P = 0.012; HR, 2.234; 95% CI, 1.192–4.187 and P = 0.040; HR, 1.729; 95% CI, 1.024–2.918, respectively). Although the same trend was observed for 5-year survival, the results did not reach statistical significance (P = 0.134; HR, 1.450; 95% CI, 0.892–2.355).

#### Significance Of SNP Rs7897947 In Clinical Outcome Of Chemotherapy-Treated Patients

SNP rs7897947 was also associated with the OS of patients who received first-line chemotherapy (n = 69) independently of the disease stage. In particular, patients with the TT genotype displayed worse 2- and 3-year survival rates than G allele carriers which persisted after FDR analysis (Fig. 2D; P = 0.006; HR, 2.576; 95% CI, 1.309–5.068, q = 0.018 and P = 0.014; HR, 2.064; 95% CI, 1.161–3.668, q = 0.021 respectively). A similar trend was also detected for 5-year OS, but was not statistically significant (Fig. 2D, P = 0.093; HR, 1.566; 95% CI, 0.928–2.643).

#### Correlations Of SNP Rs7897947 With Clinicopathological Parameters

SNP rs7897947 was not associated with disease stage at presentation (P = 0.519) and smoking history was similar between stages I and II and stages III and IV (P = 0.978). Moreoever, rs7897947 genotype was not associated with age, gender, primary location, histological subtype, grade, maximum diameter, lymph node infiltration, regional relapse or metastatic progression (see Supplementary Table S1). In addition, rs7897947 genotypes (either dominant or recessive model) were not associated with cytoplasmic (P = 0.991 and P = 0.608, respectively) or nuclear protein expression (P = 0.801 and P = 0.943, respectively) or mRNA expression levels (P = 0.486 and P = 0.393, respectively).

### *NF-kB2* SNP Rs12769316 Was Associated With NSCLC Risk

Although genotype frequencies for rs12769316 were in equilibrium for healthy controls, deviations from the Hardy-Weinberg model were found for NSCLC patients (P = 0.8159 and P = 0.0003, respectively). An increased risk of NSCLC was associated with the rs12769316 minor allele A (Table 1) and in particular the homozygous genotype AA compared to the AG and GG genotypes (Table 1).

#### SNP Rs12769316 And Survival Of Stage I or II NSCLC Patients

**Figure 3:**
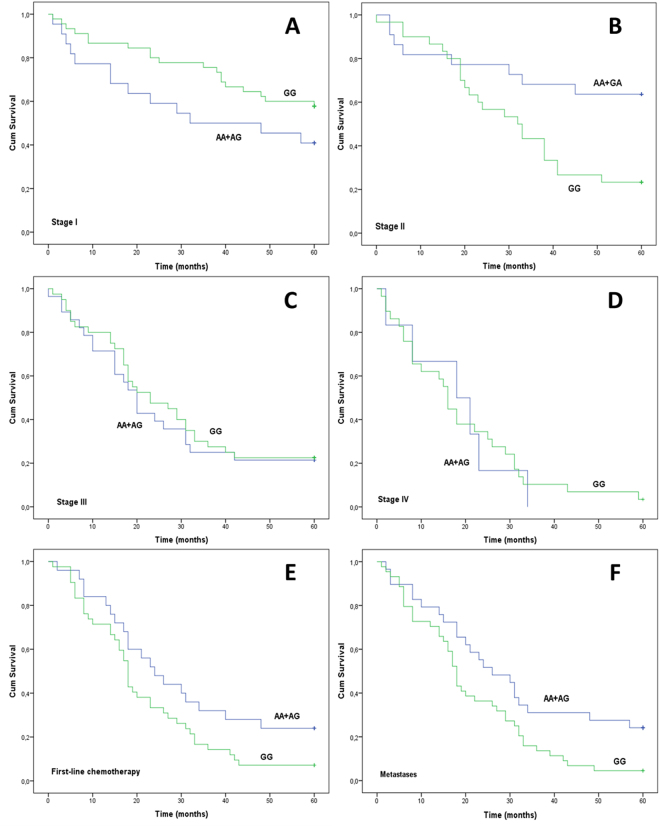
Overall survival (OS) and rs12769316 genotypes. Kaplan-Meier plot of OS of NSCLC patients according to the dominant model for the A allele. (**A**) Stage I patients, (**B**) stage II patients, (**C**) stage III patients, (**D**) stage IV patients, (**E**) patients who received first-line chemotherapy, (**F**) patients who developed metastases (brain, liver, bones, adrenals).

Rs12769316 genotypes were not associated with 2-, 3- and 5-year survival (P = 0.632; HR, 1.101; P = 0.967; HR, 0.993 and P = 0.513; HR, 0.896, respectively). However, when stage was taken into consideration, associations between survival of stage I or II patients and rs12769316 were observed (Fig. 3A,B), which persisted upon FDR correction. Particularly, the A allele was related to a worse 3-year survival in stage I patients in both univariate and multivariate (adjusting for age, sex, primary site and histological subtype) analysis (P = 0.031; HR, 2.426; 95% CI, 1.050–5.603 and P = 0.047; HR, 2.353; 95% CI, 1.012- 5.467, respectively). On the contrary, stage II G homozygotes displayed a worse 5-year survival compared to A allele carriers in univariate analysis (P = 0.006; HR, 3.049; 95% CI, 1.298–7.165) and in multivariate analysis (P = 0.029; HR, 3.022; 95% CI, 1.117–8.17), whereas OS of stage III and IV patients was unaffected by rs12769316 (Fig. 3C,D, P = 0.687; HR, 1.116; 95% CI, 0.646–1.929 and P = 0.874; HR, 1.072; 95% CI, 0.439–2.617).

Among the patients who received first-line chemotherapy (n = 67), independent of the disease stage, a trend was noted between the rs12769316 genotypes and 5-year OS, but was not statistically significant (Fig. 3E, P = 0.062; HR, 0.600; 95% CI, 0.345–1.043). Additionally, amongst patients who developed liver, brain, bone and adrenal gland metastases, A allele carriers had better 5-year survival compared to G homozygotes even after FDR analysis (Fig. 3F, P = 0.020; HR, 0.548; 95% CI, 0.324–0.925).

#### SNP Rs12769316 Was Associated With NF-kB2 Expression

Cytoplasmic but not nuclear NF-kB2 protein levels were associated with the presence of rs12769316 with G allele carriers having higher cytoplasmic expression than AA homozygotes (Supplementary Figure S1A, P = 0.048). In addition, GG homozygotes were found to have lower *NF-kB*2 mRNA levels compared to A allele carriers (Supplementary Figure S1B, P = 0.008).

#### *NF-kB2* SNP rs12769316 Was Associated With Disease Stage And Tumor Maximum Diameter

According to the dominant model for allele A, rs12769316 was associated with disease stage (P = 0.037) with G homozygosity being correlated with a higher frequency of metastatic disease (stage IV) compared to A allele carriers (P = 0.010).

In addition, rs12769316 genotypes were associated with the maximum diameter of the primary tumor lesions. Using a three-tier system (≤3.5 cm vs >3.5 cm to ≤6.0 cm vs >6.0 cm), NSCLC patients with tumors bigger than 6 cm maximum diameter were more frequently heterozygotes (P = 0.025).

Rs12769316 was independent of age, gender, primary location, histological subtype, grade, lymph node infiltration, smoking history and relapse rate (see Supplementary Table S2). A statistically significant association was noted between rs12769316 and the development of brain metastases (P = 0.048), which, however, requires further evaluation due to the small number of patients in each genotypic subgroup.

### Rs11574852 Was Associated With Age And Regional Relapse

Rs11574852 was associated with age and regional relapse (P = 0.038 and P = 0.013, respectively) (see Supplementary Table S3). No significant correlation was found between rs11574852 and NSCLC risk, gender, primary location, histological subtype, grade, lymph node infiltration, maximum diameter, regional relapse or metastasis development (Table S3). Additionally, no relation was found between this SNP, cytoplasmic (P = 0.191) or nuclear NF-kB2 protein expression (P = 0.857) as well as mRNA levels (P = 0.222).

## Discussion

Over the last decades, a progressive understanding of the molecular biological mechanisms behind NSCLC development has led to the realization that NSCLC should no longer be considered only as a single, uniform disease but rather as a heterogeneous entity^30^. It is therefore imperative to focus on a more precise and personalized treatment management of NSCLC patients in order to improve significantly the response to treatment and thus the survival outcome^31^. In this vein, the SNP profile of specific genes can be potentially used not only for identifying high-risk individuals, but also for predicting response to therapy and survival outcome, aiming to achieve a more personalized treatment approach^14,32^.

In this study, three SNPS of the *NF-kB2* gene were evaluated in NSCLC patients and healthy controls through monitoring of their association with *NF-kB2* expression, clinicopathological parameters and clinical outcome of the patients. Two of these (rs12769316 and rs7897947) appear to have a role in NSCLC.

Rs12769316 was found to be associated with NF-kB2 protein and mRNA expression which could be justified by its location, upstream of the NF-kB2 transcription start site and on the promoter region of the *NF-kB2* gene, within a binding site for the CCCTC-binding transcription factor (CTCF)^33^. In addition, the internet-based PROMO software predicted more transcription factor binding sites in the genomic region of rs12769316^34^. The possible functional role of rs12769316 in the transcriptional regulation of *NF-kB2* gene was also predicted from the internet-based bioinformatic tool F-SNP^35^. The second polymorphism (rs7897947) was not related to NF-kB2 protein and mRNA expression although was related to other aspects of NSCLC as is discussed below. It is located on the 8^th^ intron of the *NF-kB2* gene, but nothing is known regarding its possible function. The web-based tool SNPinfo indicated a lack of association of this SNP with splicing or a possible miRNA binding site^36^. On the other hand, using the variant effect predictor (VEP), we noted that rs7897947 was located on the non-coding RNA transcript ENST00000467116 which retains intron 8 and through which it is possible to function^37^.

Our study further revealed an association of rs12769316 and rs7897947 with NSCLC risk. Notably, minor allele A of rs12769316 and homozygotes TT of rs7897947 were associated with a higher risk of NSCLC development. The role of these two SNPs has also been studied in hematological malignancies, especially in bortezomib-based treatment of patients with multiple myeloma where disease development was independent of rs7897947 while a trend was noted for an association with rs12769316^29^.

Another intriguing finding of the present study is the correlation of rs7897947 and rs12769316 with OS. Notably, OS was significantly better for rs7897947 G allele carriers, especially for stages III and IV patients compared to homozygotes TT, which persisted after FDR corrections. This observation in conjunction with the association of rs7897947 with the OS of patients receiving first-line chemotherapy and with the development of distant metastases may be indicative of its role in systemic treatment. However the association of rs7897947 with a higher risk of NSCLC possibly reflects a more fundamental and pivotal role of this SNP in disease initiation and progression. Supportive to this is also our observation that the T allele frequency was higher in stages III and IV compared to stages I and II.

Interestingly, rs12769316 was also associated with OS. Notably, the presence of the A allele of rs12769316 was related to decreased OS rates, especially for stage I patients whereas surprisingly the opposite was noted for stage II patients. The association of rs12769316 with OS is also compatible with the correlation of this SNP with the risk of NSCLC development. This is not the first time that this SNP has been related to the OS of patients. Presence of the A allele has been associated with a superior OS in multiple myeloma patients treated with bortezomib-based regimens^29^. The observed differences in clinical outcome when we take into account the rs12769316 genotype, the stage and the treatment or not with first-line chemotherapy support further our notion that rs12769316 may interplay with therapeutic choices.

The possible mechanisms through which rs7897947 and rs12769316 could be implicated in NSCLC pathogenesis have not been studied with the effects of both SNPs at the molecular level remaining unknown. We have recently reported that another polymorphism (*BCL3* rs8100239) in the alternative pathway of NF-kB is associated with survival of NSCLC patients^14^, which together with the current findings perhaps suggest that SNPs in genes of this pathway infer the importance of this pathway in cancer and may have a role in lung cancer pathogenesis and/or in response to therapy.

Despite the promising results of our study, we must acknowledge some weak points. A major limitation of our study is that retrospectively collected samples (Group R) include mainly surgical specimens and therefore early stage lung cancer cases, whereas in Group P cases are almost equally distributed in the different stages. In order to minimize the effect of this point we have performed analyses not only in Group R and in Group P, but also in Group PR (analysis based on combined groups P and R) without detecting significant differences to sub-analyses. Another weak point is that immunohistochemistry was performed in a cohort almost similar to Group R, with a few stage IV cases, mainly due to the limitations in tissue availability. Larger cohorts and ideally an independent validation group would be a logical next step in order to achieve more robust conclusions.

In conclusion, we report for the first time that two (rs7897947 and rs12769316) of the three studied genetic variants of the *NF-kB2* are associated with increased risk and decreased OS outcome of patients with NSCLC. These findings further support our previous work regarding the role of the alternative pathway of NF-kB in NSCLC^15^. Functional studies will shed light on the molecular mechanisms through which these SNPs can affect the development and therapeutic response of NSCLC.

## Methods

### Study Design, Population and Data Collection

This study was approved by the Committee on Research and Ethics as well as the Scientific Committee of the University Hospital of Patras, Greece and was designed according to the ethical guidelines of the Helsinki Declaration (2013)^38^. The participants took part voluntarily with written informed consents obtained from all.

To perform the present study, we used a two-stage design in order to assess SNPs in relation to clinicopathological data, therapeutic choices, and clinical outcome (Fig. 1C). Firstly, three SNPs were evaluated in the *NF-kB2* gene region (rs7897947, rs11574852, rs12769316) in a retrospectively-collected group, followed by validation of our preliminary results in a second prospectively collected patient cohort, with a statistical analysis performed in each subgroup. In order to enhance the association and to achieve more robust statistical results, we performed a meta-analysis of the retrospectively and prospectively groups, the results of which are presented here.

Clinicopathological data, disease outcome, and vital status after a 60 month observation period were obtained from pathology reports, from medical records or through direct communication with the patients. Characteristics of the study population and relevant clinicopathological information are included in Supplementary Table S4.

A total cohort of 272 previously untreated patients of all ages with histopathologically or cytologically confirmed NSCLC (squamous carcinoma or adenocarcinoma or large cell carcinoma) was enrolled for this study. For the retrospectively collected group (Group R), 148 tumor-adjacent, non-malignant and macroscopically normal tissue specimens were retrospectively collected from the archives of the Department of Pathology of the University Hospital of Patras, Greece. Additionally, peripheral blood samples were prospectively collected from 2008 to 2010 from 124 newly diagnosed NSCLC patients (Group P). All cases were diagnosed and medically managed at the Division of Medical Oncology and the Department of Cardiothoracic Surgery at the University Hospital of Patras between 2004 and 2015. Standard-of-care treatment options were applied to all patients of the study according to the disease stage, the comorbidities, and the performance status following the current treatment guidelines. As case-exclusion criteria from our study, we considered the uncertain histology, the non-Greek Caucasian ethnic origin and the previous cancer-related therapeutic managements.

During the same period, peripheral blood specimens from 279 healthy control donors (Group H) were collected in the context of annual medical check-up. Groups of controls and patients were age- and sex-matched (Supplementary Table S5). The similarity of the genetic backgrounds was ensured by recruiting only persons with Greek Caucasian origin. Exclusion criteria included cancer- or any disease-related medical history and cancer family history and known gene mutations which predispose to cancer. The same cohort was used in a previous association study, the results of which were recently published^14^.

### Selection Οf SNPs

Literature (PubMed, Google Scholar) was reviewed by using suitable keywords (SNPs, NF-kB2, cancer, NSCLC, lung cancer, risk factor) for the selection of particular SNPs found within the *NF-kB2* gene that had been previously assessed regarding their role in cancer. SNP rs12769316 had been found to be correlated with overall survival (OS) of patients with multiple myeloma^29^. In addition, tagging SNPs rs7897947 and rs11574852 were selected through the use of the internet-based tools “Tagger” and “SySNPs for the CEU population^39,40^. The cut-off for minor allele frequency was determined at 5% and the coefficient of determination r^2^ = 0.8 was captured. Genomic location, minor allele frequencies (MAF) and other specific characteristics of SNPs are presented in Supplementary Table S6.

### DNA Isolation

Genomic DNA was extracted from whole blood samples of 124 cancer patients (Group P) and 279 healthy donors (Group H) using the commercial kit “QIAmp mini blood kit” (QIAGEN Ltd., Crawley, UK). Furthermore, DNA was also isolated from 148 FFPE, tumor adjacent, non-malignant tissue specimens (Group R) using the “QIAamp DNA FFPE Tissue kit” (QIAGEN Ltd., Crawley, UK). DNA was stored at −20 °C until required.

### Genotyping

Genotyping was performed for the selected polymorphisms, blinded to the case status, by a real-time PCR technique, on the basis of detection differences in the melting temperatures of the products (*T*_m_) as described by Wang *et al*.^41^. A GC-tail was introduced at the end of one of the two allele-specific primers that we designed, making the discrimination of alleles feasible by producing differences in amplicon *T*_m_, further identified using SYBR Green I and melting curve analysis. Real-time PCRs were performed using a MX3000p PCR cycler (Stratagene, La Jolla, CA, USA). Primers were tested for specificity with BLAST (http://blast.ncbi.nlm.nih.gov/) and synthesized by VBC-GENOMICS Bioscience Research GmbH (Vienna, Austria). Reactions were performed in duplicate using the KAPA SYBR FAST Master Mix (KAPA BIOSYSTEMS, Woburn, MA, USA). No template controls were included in each PCR reaction. PCR conditions, primers sequences and relative concentrations are available upon request.

### Sequencing

Due to the observed deviation from the Hardy-Weinberg Equilibrium (HWE) of the cancer patients genotypes and in order for the results of the melting temperature (*T*_m_)-shift SNP genotyping analysis to be confirmed, further validation was achieved through sequencing of several samples, representative of all three genotypes. The sequences of the primers and PCR conditions that we used for sequencing can also be provided upon request. Sequencing was performed at Cemia SA (University of Thessaly, Greece). All obtained sequences were in agreement with our genotyping results.

### Immunohistochemical Analysis

Immunohistochemical staining of NF-kB2 was assessed in 151 NSCLC tumor samples (see Supplementary Table S7) and representative, tumor-adjacent, normal tissue specimens, following standard streptavidin-biotin-peroxidase complex procedures. These samples were selected based on availability of full clinical information and adequacy of tissue specimen. Four μm sections from FFPE samples were deparaffinised in xylene and rehydrated in a series of graded alcohols. Next, the sections were pre-treated in a microwave oven and peroxidase activity was blocked with 3% hydrogen peroxide for 20 min, followed by incubation with an appropriate protein blocking solution. A mouse monoclonal anti-NF-kΒ2 antibody (dilution 1:500, clone: C-5 sc-7386, Santa Cruz, USA) was used as the primary antibody. Detection was performed using the Envision detection kit (Dako Cytomation Co., Glostrup, Denmark) according to the manufacturer’s instructions. Diaminobenzidine (DAB) was used as the chromogen for visualization. Sections were counterstained with Harris’ hematoxylin solution, dehydrated and mounted. To test the specificity, the procedure was repeated in consecutive sections, substituting the anti-NF-kB2 antibodies with protein blocking solution.

### Evaluation Of Immunohistochemistry

For the evaluation of the stained slides the same procedure was followed as previously described^15^. One pathologist (H.P.) and one investigator (F.D.) assessed all slides independently, blinded to the case. Histological type and tumor grade were confirmed according to the 2004 WHO classification of lung tumours^42^. The intensity and distribution of the NF-kB2 signal were the parameters used to estimate NF-kB2 expression. The total score for each slide was calculated as the sum of intensity (0–3) and distribution (0–3), forming a semiquantitative scoring system (between 0 and 6). Cases with staining in >10% of cells were considered positive. Using the 33^rd^ and 66^th^ percentiles as cut-offs, NF-kB2 expression in cancer cells was categorized into three groups (high vs. medium vs. low). For each tissue section, the more representative areas were selected using low-power fields (magnification 10x). Accurate quantification was performed on high power fields (magnification 40x).

### Gene expression analysis of *NF-kB2* by Quantitative Real-Time PCR (qRT-PCR)

By qRT-PCR, we measured NF-kB2 gene expression. RNA isolation from 59 patients was performed using the commercially available kit NucleoSpin® totalRNA FFPE Kit (MACHEREY-NAGEL, GmbH & Co., Düren Germany) followed by reverse transcription and cDNA synthesis based on SuperScript III First-Strand Synthesis System (Life Technologies, Carlsbad, CA, USA) according to the protocol. Specific primers for *NF-kB2* and *IPO8* (Importin 8, used as a reference gene) genes were designed using OligoAnalyzer 3.1 according to the sequences given in NCBI (http://www.ncbi.nlm.nih.gov/). Primers were synthesized by IDT. Both primer sequences as well as reaction conditions will be provided upon request. The PCRs were performed in triplicate in an MX3000p (Stratagene, La Jolla, CA, USA) cycler. LinRegPCR software was used in order to quantify detected signals and normalize them to the levels of IPO8^43^.

### Statistical Analysis

In order to test the deviation of our population from the HWE, a standard χ^2^-test was used. Differences in the distribution of genotypes between patients and healthy controls were also calculated using the χ^2^ test. To evaluate associations between genotypes and NSCLC susceptibility, logistic regression and odds ratios (ORs) were used based on an unconditional model. Possible associations between genotypes and clinicopathological parameters of the tumors or expression were evaluated using the Kruskal-Wallis or the Mann-Whitney tests for ordinal variables, χ^2^ test for nominal variables, and T test for continuous variables. In some cases, to assess the possible relations between genotypes and NF-kB2 expression, a logistic regression analysis was performed.

Overall survival time was determined as time from the date of surgery or from the date of histological or cytological diagnosis until the date of confirmed death or last follow-up. Univariate analysis was performed estimating survival rates with the Kaplan–Meier method and then compared with the log-rank test. Cox Proportional Hazards models and Hazard Ratios (HRs) were calculated in order for a multivariate survival analysis to be conducted using age, sex, primary site, disease stage and histological subtype as co-factors. False discovery rate (FDR) analysis was performed using the excel spreadsheet calculator available at the Handbook of Biological Statistics by John H. McDonald (http://www.biostathandbook.com/multiplecomparisons.html). Statistical significance persisted after FDR analysis for all P values < 0.05 reported, unless otherwise indicated in the text. Statistical analyses were performed using the IBM SPSS Statistics software for Windows, Version 21.0 (Armonk, NY: IBM Corp). For all comparisons, statistical significance was defined as *P* < 0.05 and all statistical tests were two-sided.

## Electronic supplementary material

Supplementary file
