# Supplementary material for: NF-kB2 Genetic Variations are Significantly Associated with Non-Small Cell Lung Cancer Risk and Overall Survival
Source: Sci Rep. 2018 Mar 27;8:5259. doi: 10.1038/s41598-018-23324-3 (PMC5869671; doi:10.1038/s41598-018-23324-3)
Supplement: Supplementary file 1 — Supplementary file [file 41598_2018_23324_MOESM1_ESM.pdf]

NF-kB2 GENETIC VARIATIONS ARE SIGNIFICANTLY ASSOCIATED WITH NON-SMALL CELL LUNG CANCER

RISK AND OVERALL SURVIVAL

[1]Foteinos-Ioannis D. Dimitrakopoulos, [1]Anna G. Antonacopoulou, [1]Anastasia E. Kottorou, [2]Stella Maroussi, [3]Nikolaos Panagopoulos, [1]Ioulia Koukourikou, [4]Chrisoula Scopa, [5]Melpomeni Kalofonou, [1]Angelos Koutras, [1]Thomas Makatsoris, [6]Helen Papadaki, [3]Dimitrios Dougenis, [7]Malcolm Brock, [1*]Haralabos P. Kalofonos

[1]Molecular Oncology Laboratory, Division of Oncology, Department of Internal Medicine, Medical School, University of Patras, Patras, Greece

[2] "G. Gennimatas" General Hospital of Athens, Neurology Department, Athens, Greece

[3]Department of Cardiothoracic Surgery, Medical School, University of Patras, Patras, Greece

[4]Department of Pathology, Medical School, University of Patras, Patras, Greece

[5]Institute of Biomedical Engineering, Imperial College London, London, United Kingdom

[6]Department of Anatomy, Medical School, University of Patras, Patras, Greece

[7]Division of Thoracic Surgery, Department of Surgery, Johns Hopkins University School of Medicine, Baltimore, MD, USA

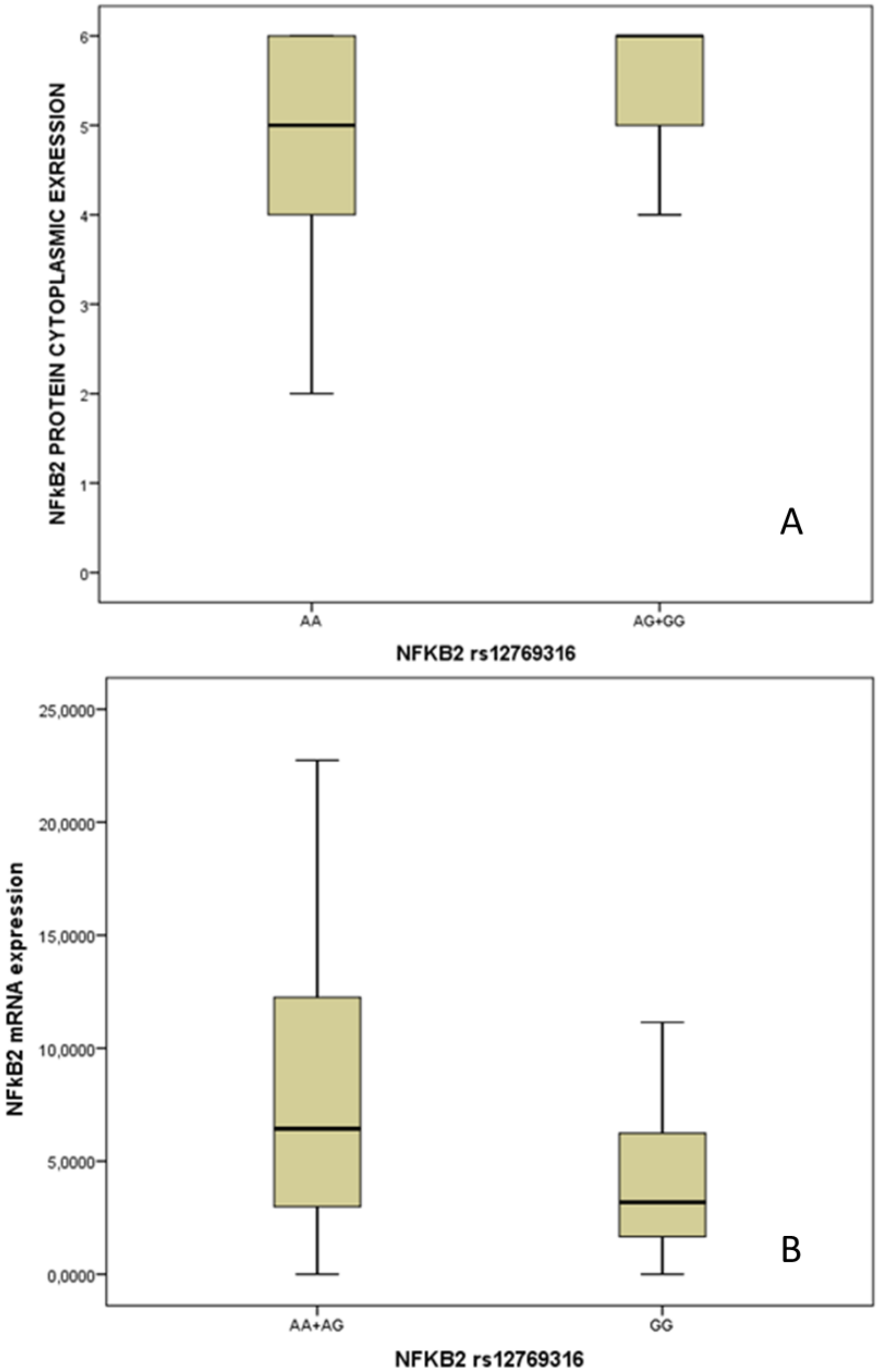

Supplementary Figure 1. NF-kB2 expression related to rs12769316 dominant model (AA vs AG+GG) in protein (A) and mRNA (B) level.

Clinicopathological characteristics	*Patients* *n* (%)	*Genotypes*		*P-value*
		TT	GG+GT	
**Total**	272 (100)			
Genotyped	264 (97.1)	145 (53.3)	119 (43.8)	-
NA	8 (2.9)	-	-	
**Age (years)** Mean (range)	65 (40-84)			
Genotyped	264 (100)			
<65	102 (38.6)	56 (21.2)	46 (17.4)	0.969
>=65	161 (61.0	88 (33.3)	73 (27.7)	
NA	1 (0.4)	1 (0.4)	0 (0.0)	
**Gender**				
Genotyped	264 (100)			
Male	242 (91.7)	135 (51.1)	107 (40.5)	0.351
Female	22 (8.3)	10 (3.8)	12 (4.5)	
**Smoking (pack-years)**				
Genotyped	264			
Cases	108 (41.0)	57 (21.6)	51 (19.4)	0.545
Mean (range)	86.30 (10-200)	83.93 (10-200)	88.94 (20-165)	
NA	156 (59.1)	88 (56.4)	68 (25.8)	
**Primary location**				
Genotyped	264 (100)			
Left lung	116 (44.0)	65 (19.3)	51 (19.3)	0.575
Right lung	139 (52.6)	73 (27.7)	66 (25.0)	
NA	9 (3.4)	7 (2.7)	2 (0.7)	
**Histology**				
Genotyped	264 (100)			
Squamous	128 (48.5)	78 (29.5)	50 (19.0)	
Adenocarcinoma	113 (42.8)	54 (20.5)	59 (22.3)	0.116
Large carcinoma	14 (5.3)	7 (2.7)	7 (2.7)	
NA	9 (2.4)	6 (2.3)	3 (1.1)	

**Stage**				
Genotyped	264 (100)			
I	75 (28.4)	44 (16.7)	31 (11.7)	
II	63 (23.9)	30 (11.4)	33 (12.5)	
III	75 (28.4)	43 (16.3)	32 (12.1)	0.519
IV	38 (14.4)	19 (7.2)	19 (7.2)	
NA	13 (4.9)	9 (2.4)	4 (1.5)	
**Grade**				
Genotyped	264 (100)			
I	7 (2.7)	6 (2.3)	1 (0.4)	
II	102 (38.6)	54 (20.5)	48 (18.1)	0.241
III	104 (39.4)	57 (21.6)	47 (17.8)	
NA	51 (19.3)	28 (10.6)	23 (8.7)	
**Maximum diameter (cm)**				
Genotyped	264 (100)			
Cases (%)	219 (83.0)	117 (44.3)	102 (38.6)	0.425
Mean (range)	4.84 (0.70-21.00)	4.88 (0.70-18.00)	4.88 (1.00-21.00)	
NA	45 (17.0)	28 (10.6)	17 (6.4)	
**Lymph node infiltration**				
Genotyped	264 (100)			
No	109 (41.30	59 (22.3)	50 (19.0)	0.838
Yes	93 (35.2)	49 (18.6)	44 (16.6)	
NA	62 (23.50	37 (14.0)	25 (9.5)	
**Metastasis (adrenals)**				
Genotyped	264 (100)			
No	45 (17.0)	26 (9.8)	19 (7.20	0.872
Yes	18 (6.8)	10 (3.8)	8 (3.0)	
NA	201 (76.1)	109 (41.3)	92 (34.8))	
**Metastasis (liver)**				
Genotyped	264 (100)			
No	43 (16.3)	25 (9.5)	18 (6.8)	0.899
Yes	23 (8.7)	13 (4.9)	10 (3.8)	
NA	198 (75.0)	102 (38.6)	91 (34.5)	

**Metastasis (brain)**				
Genotyped	264 (100)			
No	43 (16.3)	24 (9.1)	19 (7.2)	0.210
Yes	36 (13.6)	15 (5.7)	21 (8.0)	
NA	185 (70.0)	106 (40.2)	79 (29.9)	
**Metastasis (bone)**				
Genotyped	264 (100)			
No	38 (14.4)	21 (8.0)	17 (6.4)	0.794
Yes	43 (16.3)	25 (9.5)	18 (6.8)	
NA	183 (69.3)	99 (37.5)	84 (31.8)	
**Metastasis (adrenals-liver-brain-bone)**				
Genotyped	264 (100)			
No	16 (6.1)	10 (3.8)	6 (2.3)	0.406
Yes	84 (31.8)	43 (16.3)	41 (15.5)	
NA	164 (62.1)	129 (48.9)	35 (13.2)	
**Survival (2 years)**				
Genotyped	264 (100)			
Dead	115 (43.5)	75 (28.4)	40 (15.2)	**0.012**
Alive	142 (53.8)	69 (26.1)	73 (27.7)	
NA	7 (2.7)	1 (0.4)	6 (2.3)	
**Survival (3 years)**				
Genotyped	264 (100)			
Dead	147 (55.7)	91 (34.5)	56 (21.2)	**0.033**
Alive	107 (40.5)	53 (20.0)	54 (20.5)	
NA	10 (3.8)	1 (0.4)	9 (3.4)	
**Survival (5 years)**				
Genotyped	264 (100)			
Dead	174 (65.9)	104 (39.4)	70 (2.7)	0.062
Alive	80 (30.3)	40 (15.15)	40 (15.15)	
NA	10 (3.8)	1 (0.4)	9 (3.4)	

Supplementary Table S1. Relationships between clinicopathological variables and *NF-kB2* rs7897947 genotypes. Abbreviations: NA, data not available or unknown.

Clinicopathological characteristics	Patients<br>*n* (%)	*Genotypes*		*P-value*
		GG	AA+AG	
**Total**	272 (100)			
Genotyped	242 (89.0)	160 (58.8)	82 (30.2)	-
NA	30 (11.0)	-	-	
**Age (years)** Mean (range)	65 (40-84)			
Genotyped	242 (100)			
<65	93 (38.4)	58 (24.0)	35 (14.4)	0.348
>=65	148 (61.2)	101 (41.8)	47 (19.4)	
NA	1 (0.4)			
**Gender**				
Genotyped	242 (100)			
Male	220 (90.9)	144 (59.5)	76 (31.4)	0.492
Female	22 (9.1)	16 (6.6)	6 (2.5)	
**Smoking (pack-years)**				
Genotyped	242 (100)			
Cases	98 (40.5)	62 (25.6)	36 (14.9)	0.350
Mean (range)	86.30 (10-200)	87.02 (10-165)	85.89 (20-200)	
NA	144 (59.5)	98 (40.5)	46 (19.0)	
**Primary location**				
Genotyped	242 (100)			
Left lung	103 (42.7)	68 (28.1)	35 (14.5)	0.151
Right lung	131 (54.1)	86 (35.5)	45 (18.6)	
NA	8 (3.3)	6 (2.5)	2 (0.8)	
**Histology**				
Genotyped	242 (100)			
Squamous	116 (47.9)	73 (30.2)	43 (17.8)	
Adenocarcinoma	107 (44.2)	73 (30.2)	34 (14.0)	0.430
Large carcinoma	10 (4.2)	5 (2.1)	5 (2.1)	
NA	9 (3.7)	9 (3.7)	-	

**Stage**				
Genotyped	242 (100)			
I	67 (27.7)	45 (18.6)	22 (9.1)	
II	52 (21.5)	31 (12.8)	21 (8.7)	**0.037**
III	71 (29.3)	42 (17.4)	29 (11.9)	
IV	39 (16.1)	33 (13.6)	6 (2.5)	
NA	13 (5.4)	9 (3.7)	4 (1.7)	
**Grade**				
Genotyped	242 (100)			
I	7 (2.9)	4 (1.7)	3 (1.2)	
II	92 (38.0)	64 (26.4)	28 (11.6)	0.362
III	95 (39.3)	57 (23.6)	38 (15.7)	
NA	48 (19.8)	35 (14.4)	13 (5.4)	
**Maximum diameter (cm)**				
Genotyped	242 (100)			
Cases (%)	198 (81.8)	125 (51.7)	73 (30.1)	**0.031**
Mean (range)	4.72 (0.70-14.00)	4.44 (0.70-11.00)	5.20 (1.00-14.00)	
NA	44 (18.2)	35 (14.5)	9 (3.7)	
**Lymph node infiltration**				
Genotyped	242 (100)			
No	98 (40.5)	58 (24.0)	40 (16.5)	0.358
Yes	82 (33.9)	54 (22.3)	28 (11.6)	
NA	62 (25.6)	48 (19.8)	14 (5.8)	
**Metastasis (adrenals)**				
Genotyped	242 (100)			
No	40 (16.5)	24 (9.9)	16 (6.6)	0.448
Yes	17 (7.0)	12 (5.0)	5 (2.1)	
NA	185 (76.4)	120 (49.6)	65 (26.9)	
**Metastasis (liver)**				
Genotyped	242 (100)			
No	39 (16.1)	21 (8.7)	18 (74.4)	0.223
Yes	23 (9.5)	16 (6.6)	7 (2.9)	
NA	180 (74.4)	123 (50.1)	57 (23.6)	

**Metastasis (brain)**				
Genotyped	242 (100)			
No	38 (15.7)	23 (9.5)	15 (6.2)	0.327
Yes	35 (14.5)	25 (10.3)	10 (4.1)	
NA	169 (69.8))	112 (46.3)	57 (23.6)	
**Metastasis (bone)**				
Genotyped	242 (100)			
No	34 (14.0)	20 (8.3)	14 (5.8)	
Yes	41 (16.9)	24 (9.9)	17 (7.0)	0.980
NA	167 (69.0)	116 (48.0)	51 (21.1)	
**Metastasis (adrenals-liver-brain-bone)**				
Genotyped	242 (100)			
No	15 (6.2)	10 (4.1)	5 (2.0)	0.759
Yes	80 (33.1)	50 (21.0)	30 (12.4)	
NA	147 (60.7)	100 (41.3)	47 (19.4)	
**Survival (2 years)**				
Genotyped	242 (100)			
Dead	105 (43.4)	68 (28.1)	37 (15.3)	0.632
Alive	130 (53.7)	87 (36.0)	43 (17.8)	
NA	7 (2.9)	5 (2.1)	2 (0.8)	
**Survival (3 years)**				
Genotyped	242 (100)			
Dead	136 (56.2)	90 (37.2)	46 (19.0)	0.967
Alive	96 (39.7)	62 (25.6)	34 (14.1)	
NA	10 (4.1)	8 (3.3)	2 (0.8)	
**Survival (5 years)**				
Genotyped	242 (100)			
Dead	159 (65.7)	108 (44.6)	51 (21.1)	
Alive	73 (30.2)	44 (18.2)	29 (12.0)	0.513
NA	10 (4.1)	8 (3.3)	2 (0.8)	

Supplementary Table S2. Relationships between clinicopathological variables and *NF-kB2* rs12769316 genotypes. Abbreviations: NA, data not available or unknown.

Abbreviations: NA, data not available or unknown.

**Clinicopathological characteristics**	***Patients*** ***n* (%)**	***Genotypes***		***P-value***
		**AA**	**AC**	
**Total**	272 (100)			
Genotyped	261 (96.0)	239 (91.6)	22 (8.4)	-
NA	11 (4.0)	-	-	
**Age (years)** Mean (range)	65 (40-84)			
Genotyped	261 (100)			
<65	101 (38.7)	97 (37.2)	4 (1.5)	**0.038**
>=65	159 (60.9)	141 (54.0)	18 (6.9)	
NA	1 (0.4)	1 (0.4)	0 (0)	
**Gender**				
Genotyped	261 (100)			
Male	238 (91.2)	217 (83.2)	21 (8.0)	0.461
Female	23 (8.8)	22 (8.4)	1 (0.4)	
**Smoking (pack-years)**				
Genotyped	261 (100)			
Cases (%)	105 (39.7)	94 (36.0)	11 (4.2)	0.497
Mean (range)	86.3 (10-200)	88.41 (10-200)	79.14 (30-135)	
NA	156 (59.8)	145 (55.6)	11 (4.2)	
**Primary location**				
Genotyped	261 (100)			
Left lung	114 (43.7)	106 (40.6)	8 (3.1)	0.299
Right lung	139 (53.3)	124 (47.5)	15 (5.8)	
NA	8 (3.0)	9 (3.4)	1 (0.3)	
**Histology**				
Genotyped	261 (100)			
Squamous	126 (48.3)	117 (45.0)	9 (3.4)	
Adenocarcinoma	112 (42.9)	102 (39.1)	10 (3.8)	0.198
Large carcinoma	14 (5.4)	11 (4.2)	3 (1.2)	
NA	9 (3.4)	9 (3.4)	0 (0)	

**Stage**				
Genotyped	261 (100)			
I	73 (28.0)	67 (25.7)	6 (2.3)	
II	62 (23.8)	55 (21.1)	7 (2.7)	
III	75 (28.7)	70 (26.8)	5 (1.9)	0.758
IV	38 (14.5)	35 (13.4)	3 (1.1)	
NA	13 (5.0)	12 (4.6)	1 (0.4)	
**Grade**				
Genotyped	261 (100)			
I	7 (2.7)	7 (2.7)	0 (0.0)	
II	101 (38.7)	91 (34.9)	10 (3.8)	0.615
III	103 (39.5)	95 (36.4)	8 (3.1)	
NA	50 (19.2)	46 (17.6)	4 (1.6)	
**Maximum diameter (cm)**				
Genotyped	261 (100)			
Cases (%)	217 (83.1)	200 (76.6)	17 (6.5)	
Mean (range)	4.85 (0.70-21.0)	4.82 (0.70-21.0)	5.27 (1.90-11.00)	0.372
NA	44 (16.9)	39 (15.0)	5 (1.9)	
**Lymph node infiltration**				
Genotyped	261 (100)			
No	107 (41.0)	101 (38.7)	6 (2.3)	
Yes	92 (35.3)	81 (31.0)	11 (4.2)	0.110
NA	62 (23.7)	57 (21.8)	5 (1.9)	
**Metastasis (adrenals)**				
Genotyped	261 (100)			
No	43 (16.5)	40 (15.3)	3 (1.2)	0.591
Yes	18 (6.9)	16 (6.1)	2 (0.8)	
NA	200 (76.6)	183 (70.1)	17 (6.5)	

**Metastasis (liver)**				
Genotyped	261 (100)			
No	42 (16.1)	39 (14.9)	3 (1.2)	0.466
Yes	24 (9.2)	21 (8.0)	3 (1.2)	
NA	195 (74.7)	179 (68.6)	16 (6.1)	
**Metastasis (brain)**				
Genotyped	261 (100)			
No	41 (15.7)	37 (14.2)	4 (1.5)	0.493
Yes	36 (13.8)	34 (13.0)	2 (0.8)	
NA	184 (70.5))	168 (64.4)	16 (6.1)	
**Metastasis (bone)**				
Genotyped	261 (100)			
**No**	36 (13.8)	35 (13.4)	1 (0.4)	0.082
**Yes**	43 (16.5)	37 (14.2)	6 (2.3)	
**NA**	182 (69.7)	167 (64.0)	15 (5.7)	
**Metastasis (adrenals-liver-brain-bone)**				
Genotyped	261 (100)			
No	16 (6.2)	16 (6.1)	0 (0.0)	0.198
Yes	84 (32.2)	76 (29.1)	8 (3.1)	
NA	161 (61.7)	147 (56.3)	14 (5.4)	
**Survival (2 years)**				
Genotyped	261 (100)			
Dead	115 (44.1)	107 (41.0)	8 (31.0)	0.335
Alive	139 (53.3)	125 (47.9)	14 (5.4)	
NA	7 (2.7)	7 (2.7)	0 (0)	
**Survival (3 years)**				
Genotyped	261 (100)			
Dead	147 (56.3)	134 (51.3)	13 (5.0)	0.961
Alive	104 (39.8)	96 (36.8)	8 (3.1)	
NA	10 (3.8)	9 (3.4)	1 (0.4)	

**Survival (5 years)**				
Genotyped	261 (100)			
Dead	173 (66.3)	159 (61.0)	14 (5.3)	0.815
Alive	78 (29.9)	71 (27.2)	7 (2.7)	
NA	10 (3.8)	9 (3.4)	1 (0.4)	

Supplementary Table S3. Relationships between clinicopathological variables and *NF-kB2* rs11574852 genotypes. Abbreviations: NA, data not available or unknown.

	Retrospectively collected cases ***(Group R)***	***Prospectively collected cases*** ***(Group P)***	***Total cases*** ***(Group PR)***
**Clinicopathological characteristics**	***Cases*** ***n* (%)**	***Cases*** ***n* (%)**	***Cases*** ***n* (%)**
**Total**	***148 (100)***	***124 (100)***	***272 (100)***
**Age (years)** Median (range)	66 (40-84)	67 (41-84)	66 (40-84)
**Gender**			
Total	148 (100)	124 (100)	272 (100)
Male	137 (92.6)	111 (89.5)	248 (91.2)
Female	11 (7.4)	124 (10.5)	24 (8.8)
**Smoking (pack-years)**			
Total	148 (100)	124 (100)	272 (100)
Cases (%)	64 (43.2)	44 (35.5)	108 (39.7)
Mean (range)	87.5 (20-165)	84.55 (10-200)	86.3 (10-200)
NA	84 (56.8)	80 (64.5)	60 (60.3)
**Primary location**			
Total	148 (100)	124 (100)	272 (100)
Left lung	66 (44.6)	51 (41.1)	117 (43.0)
Right lung	82 (55.4)	64 (51.6)	146 (53.7)
NA	0 (0.0)	9 (7.3)	9 (3.3)
**Histology**			
Total	148 (100)	124 (100)	272 (100)
Squamous	85 (57.4)	46 (37.1)	131 (48.1)
Adenocarcinoma	53 (35.8)	64 (51.6)	117 (43.0)
Large carcinoma	9 (6.1)	6 (4.8)	15 (0.06)
NA	1 (0.0)	8 (6.5)	9 ((0.04)

**Stage**			
Total	148 (100)	124 (100)	272 (100)
I	56 (37.8)	23 (18.6)	79 (29.0)
II	46 (31.1)	19 (15.3)	65 (23.9)
III	43 (29.1)	34 (27.4)	77 (28.3)
IV	2 (1.4)	36 (29.0)	38 (14.0)
NA	1 (0.6)	12 (9.7)	13 (4.8)
**Grade**			
Total	148 (100)	124 (100)	272 (100)
I	5 (3.4)	2 (1.6)	7 (2.6)
II	66 (44.6)	41 (33.1)	107 (39.3)
III	67 (45.3)	40 (32.2)	107 (39.3)
NA	10 (6.7)	41 (33.1)	51 (18.8)
**Maximum diameter (cm)**			
Total	148 (100)	124 (100)	272 (100)
Cases (%)	144 (97.3)	83 (66.9)	227 (83.5)
Mean (range)	5.15 (0.70-21.0))	4.35 (1.0-10.0)	4.85 (0.70-21.0)
NA	4 (2.7)	41 (33.1)	45 (16.5)
**Lymph node infiltration**			
Total	148 (100)	124 (100)	272 (100)
No	77 (52.0)	37 (29.8)	114 (41.9)
Yes	65 (43.9)	31 (25.0)	96 (35.3)
NA	6 (4.1)	56 (45.2)	62 (22.8)
**Metastasis (adrenals)**			
Total	148 (100)	124 (100)	272 (100)
No	30 (20.3)	15 (12.1)	45 (16.5)
Yes	7 (4.7)	12 (9.7)	19 (7.0)
NA	111 (75.0)	97 (78.2)	208 (76.5))
**Metastasis (liver)**			
Total	148 (100)	124 (100)	272 (100)
No	31 (20.9)	12 (9.7)	43 (15.8)
Yes	6 (4.1)	17 (13.7)	23 (8.5)
NA	111 (75.0)	95 (76.6)	206 (75.70

**Metastasis (brain)**			
Total	148 (100)	124 (100)	272 (100)
No	31 (20.9)	12 (9.7)	43 (15.8)
Yes	9 (6.1)	27 (21.8)	36 (13.2)
NA	108 (73.0)	85 (68.5)	193 (71.0)
**Metastasis (bone)**			
**Total**	148 (100)	124 (100)	272 (100)
**No**	24 (16.20	14 (11.3)	38 (14.0)
**Yes**	16 (10.8)	27 (21.8)	43 (15.8)
**NA**	108 (73.0)	83 (66.9)	191 (70.2)
**Metastasis (adrenals-liver-brain-bone)**			
Total	148 (100)	124 (100)	272 (100)
No	10 (6.8)	6 (4.8)	16 (5.9)
Yes	35 (23.6)	50 (40.3)	85 (31.2)
NA	103 (69.6)	68 (54.8)	171 (62.9)
**Survival (2 years)**			
Total	148 (100)	124 (100)	272 (100)
Dead	54 (36.5)	65 (52.4)	119 (43.8)
Alive	92 (62.2)	54 (43.5)	146 (53.7)
NA	2 (1.3)	5 (4.1)	7 (2.5)
**Survival (3 years)**			
Total	148 (100)	124 (100)	272 (100)
Dead	73 (49.3)	78 (62.9)	151 (55.5)
Alive	70 (47.3)	40 (32.3)	110 (40.4)
NA	5 (3.4)	6 (4.8)	11 (4.1)
**Survival (5 years)**			
Total	148 (100)	124 (100)	272 (100)
Dead	86 (58.1)	93 (75.0)	179 (65.8)
Alive	57 (38.5)	25 (20.2)	82 (30.1
NA	5 (3.4)	6 (4.8)	11 (4.1)

**Relapse**			
Total	148 (100)	124 (100)	272 (100)
No	15 (10.1)	1 (0.8)	16 (5.9)
Yes	22 (14.9)	12 (9.7)	34 (12.5)
NA	111 (75.0)	111 (89.5)	222 (81.6)

Supplementary Table S4. Clinicopathological characteristics of patients of this study. NA: not available or no further specifically categorized (e.g NSCLC vs squamous histology). Abbreviations: NA, data not available or unknown.

Groups (persons)	Patients group (272)	Controls group (279)	P value
Age (years)	64.68 (40-84)	64.45 (30-95)	0.815
Gender no (males/females)	248/24	245/34	0.214

Supplementary Table S5. Demographic characteristics of the controls and patients groups.

***NF-kB2* gene location**	**Rs number**	**Base change**	**Genomic position (forward strand)**	**Genotyping success rate**	**Minor allele frequency**		
					**Controls**	**1000genomes (CEU)**	**1000genomes (TSI)**
5’ flanking region	rs12769316	G > A	10:102392994	95.06	0.153 (A)	0.187 (A)	0.159 (A)
Intron 8	rs7897947	T > G	10:102397954	99.27	0.337 (G)	0.192 (G)	0.206 (G)
Intron 20	rs11574852	A > C	10:102401718	98.53	0.042 (C)	0.051 (C)	0.065 (C)

Supplementary Table S6. SNPs information. 1000 Genomes;1000 Genomes Project, CEU; Utah Residents (CEPH) with Northern and Western European Ancestry, TSI; Toscani in Italia

	**Total cases**
**Clinicopathological characteristics**	***Cases*** ***n* (%)**
**Total**	151 (100)
**Age (years)** Median (range)	66 (40-84)
**Gender**	
Total	151 (100)
Male	139 (92.1)
Female	12 (7.9)
**Smoking (pack-years)**	
Total	151 (100)
Cases (%)	59 (39.1)
Mean (range)	87.63 (20-165)
NA	92 (60.9)
**Primary location**	
Total	151 (100)
Left lung	66 (43.7)
Right lung	85 (56.3)
NA	-
**Histology**	
Total	151 (100)
Squamous	86 (57.0)
Adenocarcinoma	54 (35.8)
Large carcinoma	10 (6.6)
NA	1 (0.6)
**Stage**	
Total	151 (100)
I	59 (39.1)
II	44 (29.1)
III	45 (29.8)
IV	2 (1.3)
NA	1 (0.7)

**Grade**	
Total	151 (100)
I	4 (2.6)
II	70 (46.4)
III	67 (44.4)
NA	10 (6.6)
**Maximum diameter (cm)**	
Total	151 (100)
Cases (%)	147 (97.4)
Mean (range)	5.09 (0.70-21.00)
NA	4 (2.6)
**Lymph node infiltration**	
Total	151 (100)
No	79 (52.3)
Yes	65 (43.0)
NA	7 (4.6)
**Metastasis (adrenals)**	
Total	151 (100)
No	29 (19.2)
Yes	7 (4.6)
NA	115 (76.2)
**Metastasis (liver)**	
Total	151 (100)
No	30 (19.9)
Yes	5 (3.3)
NA	116 (76.8)
**Metastasis (brain)**	
Total	151 (100)
No	30 (19.70
Yes	9 (6.0)
NA	112 (74.2)

**Metastasis (bone)**	
Total	151 (100)
No	23 (15.2)
Yes	17 (11.3)
NA	111 (73.5)
**Metastasis (adrenals-liver-brain-bone)**	
Total	151 (100)
No	9 (6.0)
Yes	36 (23.8)
NA	106 (70.2)
**Survival (2 years)**	
Total	151 (100)
Dead	56 (37.1)
Alive	93 (61.6)
NA	2 (1.3)
**Survival (3 years)**	
Total	151 (100)
Dead	75 (49.7)
Alive	71 (47.0)
NA	5 (3.3)
**Survival (5 years)**	
Total	151 (100)
Dead	91 (60.3)
Alive	55 (36.4)
NA	5 (3.3)
**Relapse**	
Total	151 (100)
No	14 (9.3)
Yes	23 (15.2)
NA	114 (75.5)

Supplementary Table S7. Clinicopathological characteristics and survival data of NSCLC patients studied by immunohistochemistry. Abbreviations: NA, data not available or unknown.
